# Prevalence of fatigue and perceived fatigability in older adults: a systematic review and meta-analysis

**DOI:** 10.1038/s41598-025-88961-x

**Published:** 2025-02-09

**Authors:** Ting Hu, Feiling Wang, Qiuchen Duan, Xueyang Zhao, Fen Yang

**Affiliations:** 1 Nursing Department, Sir Run Run Shaw Hospital, Zhejiang University School of Medicine, Hangzhou, China; 2 Tong Ji Hospital Tong Ji Medical College Hua Zhong University of Science & Technology, Wuhan, China; 3 Ningbo Municipal Hospital of Traditional Chinese Medicine (TCM), Affiliated Hospital of Zhejiang Chinese Medical University, Ningbo, China; 4 Hubei University of Chinese Medicine, Wuhan, China

**Keywords:** Fatigue, Frail elderly, Meta-analysis, Prevalence, Systematic review, Health care, Medical research

## Abstract

Fatigue is a common health complaint in older adults, but its prevalence varies widely among studies due to differences in populations and assessment tools. The objective of this review is to systematically evaluate the prevalence of fatigue and perceived fatigability in older adults with PRISMA 2020. Four databases—PubMed, Embase, Web of Science (WoS), and Cochrane Library—were systematically searched as of December 27, 2023. Cochrane *Q* tests and the *I*^*2*^ statistic were used using Stata16.0 to assess between-study heterogeneity. A total of 21 studies involving 17843 participants were included in this study. The prevalence of fatigue in older adults was 42.6%, and the prevalence of perceived physical fatigability and mental fatigability was 58.2% and 24.0%. Meta-analysis showed that the prevalence of perceived physical fatigability among older adults was very high. This prevalence varied with regional economic development level, age of the subjects, sample size, and representativeness of the subjects. Fatigue is a health dilemma faced by most older adults. To improve quality of life, early and regular fatigue assessment should be part of routine health screening for older adults.

## Introduction

Fatigue is defined as a subjective feeling of weakness, lack of energy, and tiredness^1^^,^^2^. Due to various of functional decline associated with aging, fatigue has become a common complaint in older adults and has a serious impact on their quality of life^3^^,^^4^. Previous studies have shown that fatigue is associated with a variety of negative health outcomes in older adults, such as reduced quality of life, functional limitation, disability, hospitalization, and death^5–7^. The population aged 60 years and older is projected to increase to 1.4 billion by 2030 and 2.1 billion by 2050, based on WHO data^8^. As the world’s population ages rapidly, the negative health consequences associated with fatigue pose a serious challenge to the care of older adults. Therefore, in the context of aging, attention to the status of fatigue has important health implications in older adults^9–11^.

The lack of an overall understanding of the prevalence of fatigue in older adults is a matter of great concern for several reasons. First, fatigue may be similar to pain in older adults, as both symptoms can act as physiological warning signs^12^. Clinically, most health care providers focus on the specific disease rather than fatigue because fatigue is often a concomitant symptom of other conditions. Second, as a subjective state of description, fatigue has specificity in older adults^13^. Different people may rate their level of fatigue at the same level, but the effect of similar ratings of fatigue levels on physical activity may vary from person to person^14^. Third, when experiencing fatigue, some older adults regard it as a normal phenomenon of aging^15^. Fourth, at present, no studies have systematically analyzed the prevalence of fatigue and fatigability in older adults. Fatigue describes the perceived state of an individual, and fatigability is a trait of an individual, characterizing the degree of fatigue they experience in relation to particular activities^12^. For example, when two people report the same level of fatigue in the past week, one is active with near-normal functional abilities, and the other is sedentary with impairments in several functional abilities. Although both experienced the same fatigue over the past week, the intensity and duration of their activities during the same period were quite different^12^. Therefore, it can be explained that the latter has a higher fatiguability compared to the former. Routine assessment of fatigue and perceived fatigability may help to identify older adults who are vulnerable to a greater than expected decline in function^16^. In other words, timely assessment and identification of fatigue dimensions will play an early warning role in health care work, avoid health risks, and improve the quality of life among older adults.

Previous studies have shown that self-report tools with clear cutoff values for scale can be used to assess fatigue and perceived fatigability in older adults and help us identify those at risk for activity limitation^17–19^. Currently, there are many studies on the prevalence of fatigue and perceived fatigability in older adults around the world. The prevalence of fatigue and perceived fatigability varied from 8.88%-88.18% in older adults^20^^,^^21^. The data from these studies were collected from different populations in different countries or regions and used different assessment tools. For example, some fatigue measurement scales are a unidimensional construct, while others specify different quality dimensions of fatigability, such as perceived physical fatigability and mental fatigability^19^. Given the important role of various measurement scales in identifying and quantifying fatigue and perceived fatigability, clarifying assessment tools for different dimensions of fatigue measurement may provide more accurate data for the assessment and classified management of fatigue in older adults^22^. Therefore, the purpose of this study was to investigate the prevalence of fatigue and perceived fatigability in older adults, in order to provide epidemiological basis for targeted intervention measures for high-risk groups.

## Methods

### Search strategy and identification of studies

The Preferred Reporting Items for Systematic Reviews and Meta-Analyses (PRISMA) guidelines were adopted for this systematic review and meta-analysis^23^. This review has been approved for registration by Prospero (registration number CRD42023495515). As of December 27, 2023, four databases—PubMed, Embase, Web of Science (WoS), and the Cochrane Library—were systematically searched by two authors (TH and ZX). The computer-based searches combined fatigue and elderly-related terms and also searched other literature such as government reports, conference proceedings, and papers for additional data. No restrictions on dates or languages were applied during the search, and Boolean terms were used to maximize the search results to ensure that all relevant articles were retrieved. The full details of the search strategy are provided in the online supplementary Materials (eMethods 1).

### Inclusion and exclusion criteria

Studies were included in this review according to the following inclusion criteria: (a) The data included in the review were from studies published in peer-reviewed journals that had been approved by the relevant ethical review bodies; (b)Fatigue in older adults is well defined and a validated fatigue or perceived fatigability measurement tool is used; (c)The prevalence of fatigue and perceived fatigability in older adults was reported accurately and clearly, either in percentages or in numbers sufficient for calculating the percentages; (d) Participants investigated were all aged 60 or above; (e)For multiple articles using data from the same population, we selected the one with the highest literature quality score.

By contrast, studies were not reviewed if they met any of four exclusion criteria: (a)These are reviews, case reports, letters or editorials; (b)Specific study populations were used, such as patients with specific diseases (such as cancer, stroke, and AIDS) or people who were fatigued due to intense work or exercise; (c)Studies that specifically refer to different mental disorders or other comorbidities. Previous research has demonstrated an independent association between mental disorders(such as major depression, social anxiety disorder, panic attacks, generalized anxiety disorder, obsessive–compulsive disorder, post-traumatic stress disorder, attention deficit hyperactivity disorder, and alcohol or substance use disorders) and fatigue^22^ (d)No studies with complete data reporting on fatigue prevalence were available even through online databases, library requests, or email correspondence with authors.

### Data extraction and quality assessment

Data from each study included in the review were extracted independently by the two main authors (TH and ZX) and were cross-validated using a standardized scale. The information extracted for each study included: author information, year of publication, country, sample size, (mean) age of participants, number and percentage of male participants, assessment instrument, outcome definition (i.e., cut-off point for a specific assessment instrument), and prevalence of fatigue. The authors who participated in data extraction for each study included in this review had been trained in standardized library data retrieval strategies.

The quality of each study ultimately included was assessed using a modified version of the Newcastle–Ottawa Scale (NOS)^24^. The NOS scale was evaluated for sample representativeness, sample size, response rate, determination of fatigue, and quality of descriptive statistical reports (eMethods 2). With a score of 0 or 1 for each dimension, studies were judged to have a low risk of bias (total score ≥ 3 points) or a high risk of bias (total score < 3 points). Complete details of the quality score for each included study are provided in eTable 1 in the Supplementary material. The table was generated by consensus among the three authors. All differences were resolved by examination and adjudication by the corresponding author (FY).

### Statistical analysis

Prevalence estimates of fatigue and perceived fatigability were calculated by aggregating study-specific estimates using a random-effects meta-analysis that accounted for between-study heterogeneity^25^. When fatigue prevalence estimates for different periods were reported in the included longitudinal studies, the overall prevalence for the entire period was used. Cochrane *Q* tests and the *I*^*2*^ statistic were used to assess between-study heterogeneity. For the Cochrane *Q* test, *p* < 0.05 represented significant heterogeneity. For *I*^*2*^ index, values ≥ 75% indicating considerable heterogeneity^26^; ^27^.

We performed a subgroup analysis of perceived physical fatigability and mental fatigability. Stratification analysis was conducted for gender, regional economic level, assessment tool and Newcastle–Ottawa Scale components^28^. We used simulations with meta-regression to analyze subgroup effects in the meta-analysis. The *Q*_*B*_ score indicated heterogeneity in the prevalence of fatigue and perceived fatigability in older adults across the subgroups. All analyses were performed with Stata 16.0.

## Results

### Study results

**Fig. 1:**
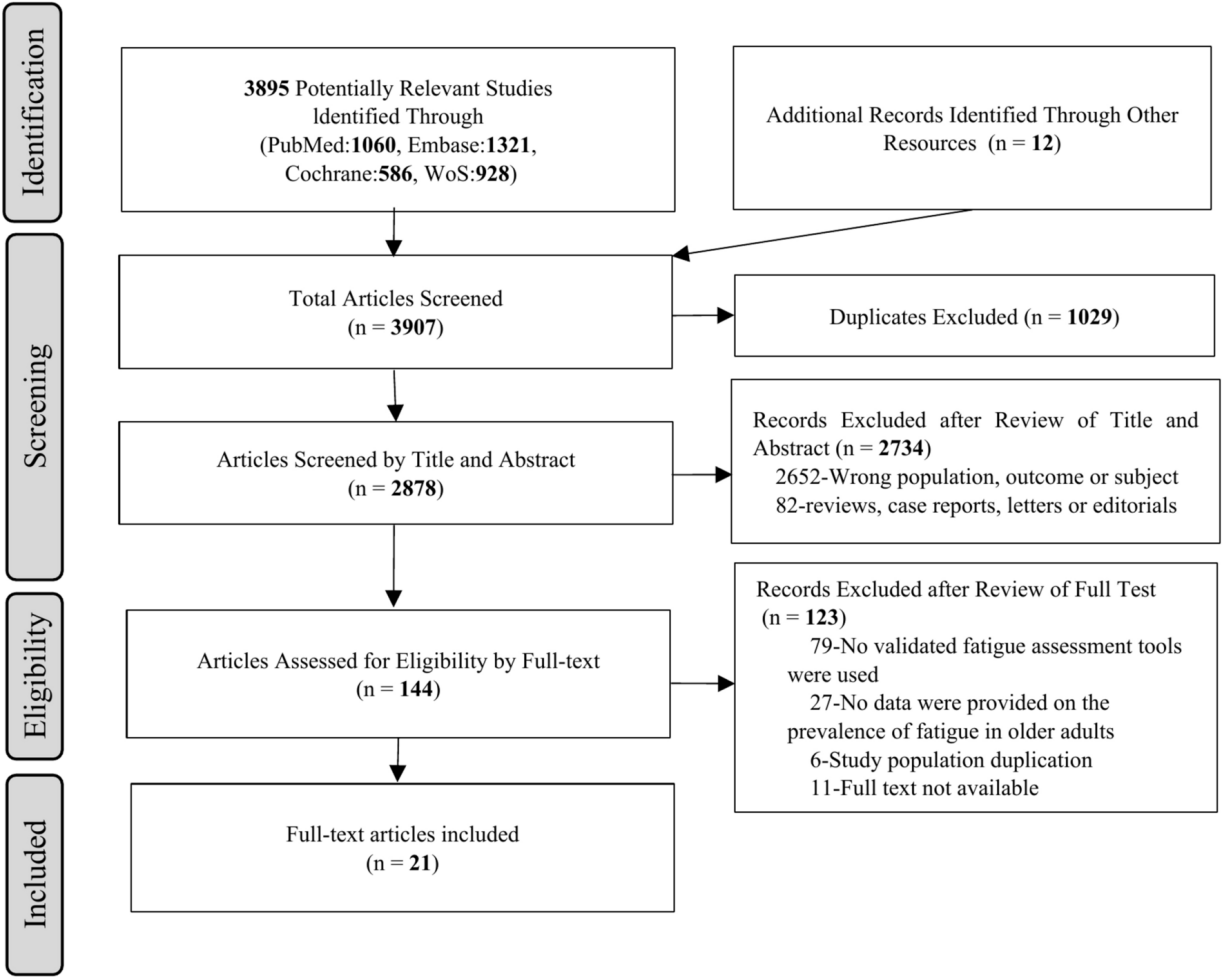
Flowchart of the study selection.

A total of 3895 studies were identified through four database searches and an additional 12 studies were identified through reference lists, leading in the selection of 3907 articles for review. After removing 1029 duplicate studies, the title and abstract of the remaining 2878 studies were reviewed. 2,652 studies were excluded because they focused at the wrong people, results, and subjects. The 82 articles were reviews, research reports, letters or editorials. Of the remaining 144 articles that were fully reviewed, 123 studies were excluded. The reasons for exclusion were that 79 studies did not use validated fatigue assessment tools, 27 studies did not provide data on the prevalence of fatigue, 6 studies had duplicate subjects, and 11 studies were not available for full text. Therefore, 21 studies were finally included for the review of this study (Fig. 1).

### Study characteristics

This systematic review and meta-analysis of 21 studies involved a total of 17,843 individuals (Table 1). According to country and regional breakdown, 11 studies were conducted in the America (11 studies were all in the United States), 2 in Asia (China, Jordan), 4 in Europe (The United Kingdom, Spain, Norway, France), one in Turkey, which spans Eurasia, and three in America and Europe (Both in the United States and Denmark).

**Table 1:** Characteristics of 21 studies included in the meta-analysis.

Study	Country	Economic level	Total No.of Participants	Average of age, mean(SD)	Men, No(%)	Assessment tool	Cut-off	No.of Participants with fatigue	NOS
Glynn et al^29^. 2022	USA and Denmark	developed country	2258	73.5(10.4)	1023(45.3)	PFS	PFS Physical ≥ 15	948	5
Alfini et al^30^. 2020	USA	developed country	382	73.1(10.3)	179(46.9)	PFS	PFS Physical ≥ 15; PFS Mental ≥ 13	Physical, n = 160; Mental, n = 83	4
Simonsick et al^18^. 2018	USA	developed country	579	73.6(NR)	271(46.8)	PFS	PFS Physical ≥ 15; PFS Mental ≥ 13	Physical, n = 238; Mental, n = 131	3
Cooper et al^31^. 2019	UK	developed country	793	Range = 68	337(42.3)	PFS	PFS Physical ≥ 15	412	5
Pérez et al^32^. 2019	Spain	developed country	79	77.2(5.0)	19(24.1)	PFS	PFS Physical ≥ 15	63	3
Egerton et al^33^. 2016	Norway	developed country	980	73.4(1.9)	509(51.9)	FSS	≥ 28	87	4
Tennant et al^34^. 2012	USA	developed country	30	80.2(4.3)	7 (23.4)	FSS	≥ 35	14	2
Hu et al^35^. 2021	China	developing country	457	84.8(5.8)	182(39.8)	PFS	PFS Physical ≥ 15	403	4
Banerjee et al^36^. 2022	USA	developed country	48	68.1(9.4)	10(20.8)	FSI	≥ 3	27	4
Malak et al^37^. 2021	Jordan	developing country	250	71.3(7.5)	120(48.0)	FACIT–Fatigue Scale	< 30	141	5
Blain et al^38^. 2021	France	developed country	1471	Range ≥ 65	485(33.0)	VAS	Female: ≥ 5; Male ≥ 4	886	4
Cho et al^39^. 2019	USA	developed country	2541	72.6(8.4)	1357(53.4)	CFQ	≥ 4	571	5
LaSorda et al^40^. 2020	USA and Denmark	developed country	2355	73.7(10.5)	1066(45.3)	PFS	PFS Physical ≥ 15	992	5
Başkurt et al^41^. 2012	Turkey	developing country	99	69.6(7.2)	50(50.5)	CIS-T	> 76	54	2
Cohen et al^42^. 2021	USA and Denmark	developed country	2361	73.6(10.5)	1068(45.2)	PFS	PFS Mental ≥ 13	585	5
Qiao et al^43^. 2022	USA	developed country	1113	84.1(3.9)	1113(100.0)	PFS	PFS Physical ≥ 15; PFS Mental ≥ 13	Physical:597; Mental:247	5
Schnelle et al^44^. 2012	USA	developed country	43	85.3(5.9)	12(27.9)	FSS	> 4	16	3
Moored et al^45^. 2021	USA	developed country	1672	84.2(4.0)	1672(100.0)	PFS	PFS Physical ≥ 15; PFS Mental ≥ 13	Physical:917; Mental:387	5
Graves et al^46^. 2021	USA	developed country	181	71.3(6.7)	38(21.0)	PFS	PFS Physical ≥ 15	111	3
Wasson et al^47^. 2019	USA	developed country	29	77.2(5.5)	4(13.8)	PFS	PFS Physical ≥ 15; PFS Mental ≥ 13	Physical: 19;	3
								Mental: 19	
Davis et al^48^. 2021	USA	developed country	122	Range ≥ 80	56(45.9)	PFS	PFS Physical ≥ 15	98	3

*CFQ* chalder fatigue questionnaire, *CIS-T* the Turkish version of checklist individual strength, *FACIT-Fatigue Scale* functional assessment of chronic illness therapy-fatigue scale, *FSI* fatigue symptom inventory, *FSS* fatigue severity scale, *PFS* Pittsburgh fatigability scale, *VAS* visual analogue scales, *NOS* Newcastle–Ottawa score, *NR* not reported.

The average number of participants in each study was 850, with a range from 29 to 2541. Thirteen studies used the Pittsburgh Fatigue Rating Scale (PFS) to assess fatigue, and three studies used the Fatigue Severity Scale (FSS). The other 5 studies used Chalder Fatigue Questionnaire (CFQ), the Turkish version of Checklist Individual Strength (CIS-T), Functional Assessment of Chronic Illness Therapy-Fatigue Scale (FACIT), The Fatigue Symptom Inventory (FSI), and Visual Analogue Scales (VAS). Eight studies addressed fatigue, 12 studies addressed perceived physical fatigability, and 6 studies addressed perceived mental fatigability.

### Study quality

When the Newcastle–Ottawa Quality Assessment criteria were used to score the quality of included studies (out of a total of 5 points), there were 2 studies with a score of 2; 6 studies with a score of 3; 4 studies with a score of 4; and 9 studies with a score of 5(the scores of individual studies are shown in eTable 1 in the Supplementary Material). All included studies used validated measurement instruments with valid cutoff scores (n = 21, 100.0%). On the other hand, 9 studies (42.8%) had suboptimal sample size representation. 7 studies (33.3%) had a sample size of less than 200 participants or convenience samples. In 4 studies (19.0%), the comparability between responders and non-responders was not satisfactory, or the response rate or characteristics of responders and non-responders were not described; Two studies(9.5%) either did not report descriptive statistics, were incomplete, or did not include appropriate dispersion measures.

### Prevalence of fatigue and perceived fatigability in older adults

**Fig. 2:**
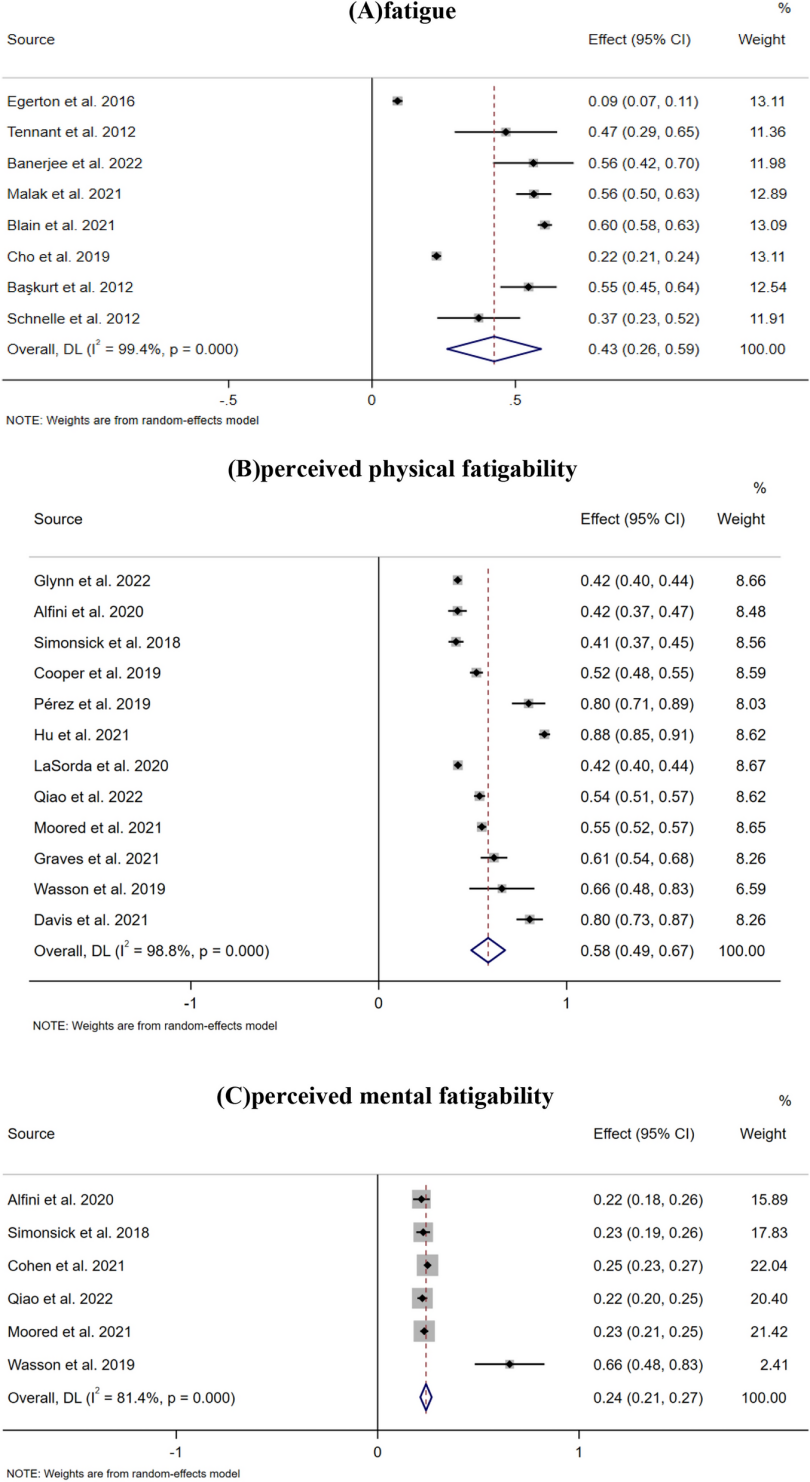
Prevalence of fatigue and perceived fatigability in older adults.

Based on the random effects model, the prevalence of fatigue in older adults was 42.6% (95% CI, 26.2%-59.0%). The prevalence of perceived physical fatigability was 58.2% (95% CI, 49.2%-67.2%), and the prevalence of perceived mental fatigability was 24.0% (95% CI, 21.2%-26.8%). There was significant heterogeneity among the studies. The *I*^*2*^ of fatigue, perceived physical fatigability and mental fatigability prevalence was 99.4%, 98.8% and 81.4%, respectively(Fig. 2).

### Subgroup analysis

To further identify the source of heterogeneity, we conducted subgroup analyses according to different characteristics of the included literature(gender, regional economic level, age, sample representativeness, sample size, comparability of respondents and non-respondents, literature quality score).

### Fatigue and perceived fatigability in older adults by gender

Nine studies provided prevalence estimates by gender(Table 2). The prevalence of fatigue was 39.1% in females and 40.7% in males. The prevalence of perceived physical fatigability among older adults was 60.3% in females and 58.8% in males, while the prevalence of perceived mental fatigability was 26.3% in females and 22.2% in males. Subgroup analysis showed no significant difference between genders in the prevalence of fatigue(*p* = 0.947), perceived physical fatigability(*p* = 0.921), or perceived mental fatigability(*p* = 0.123).

**Table 2:** Prevalence of fatigue and perceived fatigability among older adults by country or regional level of economic development, gender, and age.

Subgroups	No. of studies	Prevalence (%)	95% CI	Test for subgroup difference
Perceived Physical Fatigability				
developed country	11	55.3	46.8–63.8	*I*^*2*^ = 98.9, *τ*^*2*^ = 0.027, *p* < 0.001
developing country	1	88.2	85.2–91.1	
female	5	60.3	34.5–86.1	*I*^2^ = 99.3, *τ*^*2*^ = 0.048, *p* = 0.921
male	7	58.8	45.7–72.0	
≥ 80	2	84.6	71.4–97.9	*I*^2^ = 99.6 *τ*^*2*^ = 0.065 *P* = 0.033
< 80	2	47.4	26.0–78.9	
Perceived Mental Fatigability				
female	1	26.3	21.4–31.2	*I*^2^ = 0.02 *τ*^*2*^ < 0.001 *P* = 0.123
male	3	22.2	20.5–23.9	
Fatigue				
developed country	6	38.1	21.3–54.8	*I*^2^ = 99.2 *τ*^*2*^ = *0.036* *P* = 0.046
developing country	2	55.9	50.7–61.1	
female	3	39.1	11.0–67.2	*I*^2^ = 99.4 *τ*^*2*^ = 0.061 *P* = 0.947
male	3	40.7	4.0–77.4	

### Fatigue and perceived fatigability in older adults by regional economic level

The results of subgroup analysis showed that for countries with different levels of economic development, the prevalence of perceived physical fatigability and general fatigue among older adults was statistically significant(Table 2). The prevalence of perceived physical fatigability was 55.3% in developed countries and 88.2% in developing countries(*p* < 0.001). The prevalence of fatigue was 38.1% in developed countries and 55.9% in developing countries(*p* = 0.046).

### Fatigue and perceived fatigability in older adults by age

The pooled prevalence of perceived physical fatigability was significantly different between the older age group under 80 years and the older age group 80 years and above(*p* = 0.033) **(**Table 2**)**.

### Other sources of heterogeneity: study-level characteristics

Subgroup analyses indicated that the pooled prevalence of fatigue in older adults did not significantly vary according to study-level characteristics(Table 3). This included sample representativeness (*Q*_*B*_ = 0.1, *p* = 0.751), sample size (*Q*_*B*_ = 0.3, *p* = 0.579), comparability of respondents and non-respondents (*Q*_*B*_ = 1.7, *p* = 0.195), descriptive statistics (*Q*_*B*_ = 0.1, *p* = 0.696), and NOS score (*Q*_*B*_ = 1.7, *p* = 0.195).

**Table 3:** Prevalence of fatigue stratified by Newcastle–Ottawa Scale components.

Newcastle–Ottawa Component	No. of studies	Prevalence (%)	95%CI (%)	Q-value	*P*-value	Tau^2^	Heterogeneity *I*^*2*^ (%)
Sample representativeness							
Less	4	40.1	16.9–63.4	1108.2	< 0.001	0.054	99.4
More	4	44.9	27.8–61.8	134.2	< 0.001	0.027	95.7
Between groups				0.10	0.751		
Sample size							
< 200 participants	3	47.2	36.2–58.2	3.84	0.146	0.005	48.6
≥ 200 participants	5	40.5	19.7–61.3	1204.3	< 0.001	0.055	99.7
Between groups				0.31	0.579		
Respondent and non-respondent comparability							
Less comparable	2	52.7	44.1–61.3	0.57	0.448	< 0.001	< 0.001
More comparable	6	40.0	22.7–57.3	1206.8	< 0.001	0.045	99.5
Between groups				1.68	0.195		
Descriptive Statistics							
Less detail	1	46.7	28.8–64.5	0.15	–	< 0.001	–
More detail	7	42.0	26.7–57.2	1239.6	< 0.001	0.040	99.4
Between groups				0.15	0.696		
NOS							
< 3	2	52.7	44.1–61.3	0.57	0.448	< 0.001	< 0.001
≥ 3	6	40.0	22.7–57.3	1206.8	< 0.001	0.045	99.5
Between groups				1.68	0.195		

The prevalence of perceived physical fatigability among older adults varied significantly across strata of the components of the Newcastle–Ottawa scale (Table 4). This included representability of the sample (*Q*_*B*_ = 25.8, *p* < 0.001), sample size (*Q*_*B*_ = 7.1, *p* = 0.008), comparability of respondents and non-respondents (*Q*_*B*_ = 13.7, *p* < 0.001), and literature quality score (*Q*_*B*_ = 11.8, *p* = 0.001).

**Table 4:** Prevalence of perceived physical fatigability stratified by Newcastle–Ottawa Scale Components.

Newcastle–Ottawa Component	No. of studies	Prevalence (%)	95%CI (%)	Q-value	*P*-value	Tau^2^	Heterogeneity *I*^*2*^ (%)
Sample representativeness							
Less	5	75.9	65.7–86.1	52.6	< 0.001	0.011	96.9
More	7	46.9	42.2–51.6	127.0	< 0.001	0.004	99.6
Between groups				25.8	< 0.001		
Sample size							
< 200 participants	4	72.5	62.1–82.5	17.5	0.001	0.008	67.8
≥ 200 participants	8	52.0	41.1–62.9	807.7	< 0.001	0.025	99.4
Between groups				7.08	0.008		
Respondent and non-respondent comparability							
Less comparable	2	41.4	38.3–44.5	0.06	0.810	< 0.001	0.17
More comparable	10	61.7	51.4–72.0	872.5	< 0.001	0.026	99.0
Between groups				13.67	< 0.001		
Descriptive Statistics							
Less detail	1	41.1	37.1–45.1	< 0.001		< 0.001	
More detail	11	58.3	48.7–67.8	891.2	< 0.001	0.027	98.9
Between groups				11.8	0.001		

The prevalence of perceived mental fatigue among older adults varied significantly at the level of sample representativeness (*Q*_*B*_ = 22.8, *p* < 0.001) and sample size (*Q*_*B*_ = 22.8, *p* < 0.001) for the components of the Newcastle–Ottawa scale **(**Table 5**)**.

**Table 5:** Prevalence of perceived mental fatigability stratified by Newcastle–Ottawa Scale components.

Newcastle–Ottawa Component	No. of studies	Prevalence (%)	95%CI (%)	Q-value	*P*-value	Tau^2^	Heterogeneity *I*^*2*^ (%)
Sample representativeness							
Less	1	65.5	48.2–82.8	< 0.001		< 0.001	
More	5	23.3	22.1–24.6	4.23	0.376	< 0.001	22.2
Between groups				22.8	< 0.001		
Sample size							
< 200 participants	1	65.5	48.2–82.8	< 0.001	–	< 0.001	–
≥ 200 participants	5	23.3	22.1–24.6	4.23	0.376	< 0.001	22.2
Between groups				22.8	< 0.001		
Respondent and non-respondent comparability							
Less comparable	2	22.3	19.6–24.9	0.11	0.743	< 0.001	0.01
More comparable	4	32.4	13.8–51.0	25.6	< 0.001	0.034	99.5
Between groups				1.12	0.290		
Descriptive Statistics							
Less detail	1	22.6	19.2–26.0	< 0.001	–	< 0.001	–
More detail	5	29.8	15.7–43.9	26.54	< 0.001	0.024	99.2
Between groups				0.94	0.333		

### Assessment of publication bias

Egger’s test indicated that there was no statistically significant publication bias in studies reporting the prevalence of fatigue(*p* = 0.304) or in those reporting perceived physical fatigability(*p* = 0.188). (The funnel plots are presented in eFig. 1and eFig. 2 of the Supplementary Material.) Nevertheless, Egger’s test demonstrated significance for studies focusing on perceived mental fatigability(*p* < 0.001) (eFig. 3).

## Discussion

In this systematic review and meta-analysis, the available evidence was summarized and meta-analysis methods were used to estimate the prevalence of fatigue in older adults. A total of 21 articles were included in this study to provide estimates of the prevalence of fatigue in a total population of 17,843 older adults respondents. The overall random effect prevalence of fatigue among adults was 42.6%, indicating that approximately 2 out of every 5 older adults worldwide are affected by fatigue. Routine assessment of fatigue may help to identify older adults who are susceptible to a greater than expected decline in function^16^.

The notion of fatigability has been broadly defined to encompass both physical and mental/cognitive aspects^22^. A person’s susceptibility to fatigue (i.e., tiredness, lack of energy) in physical and mental activities is measured by perceived physical (whole-body) and mental (cognitive) fatigability^49^. Our findings revealed that perceived mental fatigability in older adults was much lower than perceived physical fatigability (24.0% (95% CI, 21.2%–26.8%) vs 58.2% (95% CI, 49.2%–67.2%)) (Fig. 2). This may be partly because recent analyses of fatigue studies suggest that perceived mental fatigability can be affected by perceived physical fatigability^47^^,^^50^. This concept is supported by earlier findings suggesting that higher perceived mental fatigability is associated with a sharp decline in physical functioning^18^^,^^50^. Secondly, in recent years, there has been relatively little research on the mental/cognitive aspects of fatigue in older adults. Therefore, there may be a possibility of publication bias.

Fatigue is common in older adults. Fatigue is not only a symptom, but also a multifactorial condition that can significantly affect the quality of life of older adults^11^^,^^51^. Therefore, the role of the healthcare worker is essential to identify, assess, and manage fatigue through a multidisciplinary approach. Subgroup analyses in this review showed that the level of economic development in different countries or regions had a significant impact on the prevalence of physical fatigue and general fatigue in older adults. The possible reason for this situation is policies in developed countries or regions that support and improve the quality of life, and the older adults in these regions tend to seek professional help to get rid of fatigue when they are tired, such as seeking multidisciplinary interventions from professionals in health care institutions and utilizing social medical insurance^52–54^. In addition to regional factors and deep-rooted cultural influences, such as religion and spirituality, other literature studies have mentioned that these factors seem to have a profound effect on enhancing fatigue tolerance and motivating individuals to adopt more positive coping strategies^11^^,^^55^. These factors can affect the perception and reporting of fatigue. However, the absence of a significant gender difference in fatigue was somewhat unexpected. In some cohort studies, the prevalence of fatigue in women was higher than that in men^18^^,^^30^^,^^31^. In addition, other methodological factors that we did not study may be important, such as issues related to fatigue response time.

Overall, our findings suggest that fatigue is common among older adults, which represents a considerable public health challenge. Fatigue can serve as a physiological warning signal, and an increase in fatigue may indicate an impending health disaster. For example, after normal activity, if one feels unprecedented fatigability, they should be cautious about whether it is caused by heart disease^56^ Weakness in bilateral arm elevation is a warning symptom of stroke^57^. A common symptom of stroke is the weakness in only one arm. When experiencing arm weakness, one arm may feel limp or numb. If you ask the person to raise both arms, the affected arm may drift downwards. However, some groups may mistakenly attribute this to fatigue^58^. Therefore, given the large impact of fatigue on health quality of life in the older adults, future management and mitigation are urgently needed to reduce the degree of fatigue in the older adults^59^. The subgroup analysis of this study highlights the need for individualized intervention strategies that take into account individual health status, lifestyle factors and specific fatigue symptoms. These strategies may include, but not limited to, nutrition adjusting, tailored according to individual ability, cognitive behavioral therapy and physical activity plan appropriate drug intervention^11,60,61^. In addition, the subgroup analysis of this study demonstrates the importance of demographic approaches, suggesting that interventions for fatigue should sensitively consider the different needs of different age, gender, and socioeconomic groups. However, despite all three studies utilizing the FSS scale, Schneider, Tennant, and Edgerton adopted different cutoff points when referencing the scale. This was done to accommodate various application contexts, balance sensitivity and specificity, and thereby enhance detection accuracy. Consequently, the current study did not conduct relevant subgroup analyses to investigate the impact of different assessment tools on fatigue prevalence.

Nevertheless, there are several strengths in this review. Firstly, we conducted a systematic analysis of articles on fatigue and perceived fatigability in older adults using a relatively comprehensive search strategy and inclusion and exclusion criteria. To our knowledge, this is the first systematic review and meta-analysis of the prevalence of fatigue in older age. Secondly, this study distinguishes between fatigue and perceived physical and mental fatigue in older adults, thereby more accurately describing the specific types of fatigue in this population. Timely capture of impending physical and cognitive decline and subclinical disease burden based on perceived fatigability assessment results^62^. Several potential limitations should be mentioned in this meta-analysis. Firstly, the high heterogeneity observed throughout the study was not explained by the relevant subgroup analysis, although this is an expected feature of an epidemiological meta-analysis. However, it must be confirmed that the conclusions drawn in this review are preliminary. Future studies need to be conducted at a higher consistency on fatigue and to explore the effects of confounding variables, to reduce the heterogeneity of research as much as possible. For example, it is noteworthy that relevant studies have shown a coexistence between fatigue and depression, potentially due to the overlapping physical, cognitive, and emotional dimensions of these symptoms. However, in the current study, due to the limited number of available literature, we did not make a specific distinction between fatigue and depression. Considering the impact of fatigue and depression on the physical and mental health of elderly individuals, it is essential that we select appropriate scales for identifying and preventing these symptoms in future assessments. Secondly, the form of fatigue assessment in the literature included in this study was self-report, and no relevant literature using performance perceived fatigability measures was included. Self-reporting biases may lead to an overestimation or underestimation of fatigue prevalence, as factors such as individual subjective perceptions, memory distortions, and societal expectations can influence the reported outcomes. Consequently, various strategies can be employed in future research to mitigate these biases, including the integration of objective physiological indicators, the adoption of standardized scales, and the conduct of multi-source information verification, in order to enhance the accuracy and reliability of assessments. Thirdly, for the 11 articles lacking full-text access, we have diligently reached out to the corresponding authors via email and thoroughly searched numerous databases for additional materials, but unfortunately, to no avail. This may have resulted in publication and selection bias to a certain extent. Fourthly, in this review, due to the limited availability of literature, we did not conduct detailed subgroup analyses of demographic data in the elderly population, such as residence (nursing home/community dwelling; urban/rural), and comorbidities/health status/functional status. We must acknowledge that these relevant demographic factors will have a significant impact on the outcomes of fatigue prevalence among the elderly.

## Conclusion

In conclusion, the results of this study illustrate the prevalence of fatigue in the older adults, which provides relevant epidemiological data to support studies on fatigue in the older adults. The prevalence of fatigue among older adults was found to be 42.6%, while the prevalence of perceived physical fatigability reached 58.2%, and the prevalence of perceived mental fatigability stood at 24.0%. The situation of fatigue among the older adults is not optimistic. Given that fatigue can to some extent represent a physiological warning system, early and regular assessment of fatigue should be part of routine health screening programs for older adults, which has important implications for improving the quality of life of older adults and for future clinical benefits. Further cohort studies are needed in the future to identify specific effective strategies to prevent and treat negative health outcomes arising from fatigue in older adults.

## Supplementary Information

Supplementary Information.

## Data Availability

The datasets used or analysed during the current study are available from the corresponding author upon reasonable request.

## References

[1] Morelli, V. Fatigue and chronic fatigue in the elderly: definitions, diagnoses, and treatments. *Clin. Geriatr. Med.* **27**, 673–686 (2011). 22062448 10.1016/j.cger.2011.07.011

[2] Su, Y. et al. Fatigue in community-dwelling older adults: a review of definitions, measures, and related factors. *Geriatr. Nurs.* **43**, 266–279 (2022). 34963072 10.1016/j.gerinurse.2021.12.010

[3] Tralongo, P., Respini, D. & Ferraù, F. Fatigue and aging. *Critical Rev. Oncol.Hematol.* **48**, S57–S64 (2003). 14563522 10.1016/j.critrevonc.2003.07.003

[4] Yu, D. S. F., Lee, D. T. F. & Man, N. W. Fatigue among older people: a review of the research literature. *Int. J. Nurs. Stud.* **47**, 216–228 (2010). 19524240 10.1016/j.ijnurstu.2009.05.009

[5] Broström, A., Wahlin, Å., Alehagen, U., Ulander, M. & Johansson, P. Sex-specific associations between self-reported sleep duration, depression, anxiety, fatigue and daytime sleepiness in an older community-dwelling population. *Scand. J. Caring Sci.* **32**, 290–298 (2018). 28574585 10.1111/scs.12461

[6] Knoop, V. et al. Fatigue and the prediction of negative health outcomes: a systematic review with meta-analysis. *Ageing Res. Rev.* **67**, 101261 (2021). 33548508 10.1016/j.arr.2021.101261

[7] Hulme, K., Little, P., Burrows, A., Julia, A. & Moss Morris, R. subacute fatigue in primary care – two sides of the story. *Br. J. Health Psychol.* **24**, 419–442 (2019). 30848557 10.1111/bjhp.12361 PMC6519220

[8] WHO. *Ageing*. (2022) Available at: https://www.who.int/health-topics/ageing#tab=tab_2 (spetember 11 August 2022).

[9] Gorasso, V. et al. The non-fatal burden of cancer in belgium, 2004–2019: a nationwide registry-based study. *Bmc Cancer.* **22**, 58 (2022). 35026995 10.1186/s12885-021-09109-4 PMC8756629

[10] Jagger, C. et al. Inequalities in healthy life years in the 25 countries of the european union in 2005: a cross-national meta-regression analysis. *Lancet.* **372**, 2124–2131 (2008). 19010526 10.1016/S0140-6736(08)61594-9

[11] Torossian, M. R., Chung, J., Mamo, S. K. & Jacelon, C. S. Examining a fatigue management model in older individuals. *Rehabil. Nurs.* **47**, 50–59 (2022). 35234405 10.1097/RNJ.0000000000000360

[12] Eldadah, B. A. Fatigue and fatigability in older adults. *Pm & R.* **2**, 406–413 (2010). 20656622 10.1016/j.pmrj.2010.03.022

[13] Romine, P. E. et al. Task-specific fatigue among older primary care patients. *J. Aging. Health.* **29**, 310–323 (2017). 26944807 10.1177/0898264316635567

[14] Kim, I. et al. Evaluation of fatigability measurement: integrative review. *Geriatr. Nurs.* **39**, 39–47 (2018). 28666548 10.1016/j.gerinurse.2017.05.014 PMC6873705

[15] Avlund, K. Fatigue in older adults: an early indicator of the aging process?. *Aging Clin. Exp. Res.* **22**, 100–115 (2010). 20440097 10.1007/BF03324782

[16] Simonsick, E. M. et al. Fatigued, but not frail: perceived fatigability as a marker of impending decline in mobility-intact older adults. *J. Am. Geriatr. Soc.* **64**, 1287–1292 (2016). 27253228 10.1111/jgs.14138 PMC4914474

[17] Glynn, N. W. et al. The Pittsburgh fatigability scale for older adults: development and validation. *J. Am. Geriatr. Soc.* **63**, 130–135 (2015). 25556993 10.1111/jgs.13191 PMC4971882

[18] Simonsick, E. M. et al. Pittsburgh fatigability scale: one-page predictor of mobility decline in mobility-intact older adults. *J. Am. Geriatr. Soc.* **66**, 2092–2096 (2018). 30315707 10.1111/jgs.15531 PMC6322394

[19] Machado, M. O. et al. Measuring fatigue: a meta-review. *Int. J. Dermatol.* **60**, 1053–1069 (2021). 33301180 10.1111/ijd.15341

[20] Hu, Y. et al. Validation of perceived physical fatigability using the simplified-chinese version of the pittsburgh fatigability scale. *Bmc Geriatr.* **21**, 336 (2021). 34039260 10.1186/s12877-021-02275-x PMC8157666

[21] Egerton, T., Riphagen, I. I., Nygård, A. J., Thingstad, P. & Helbostad, J. L. Systematic content evaluation and review of measurement properties of questionnaires for measuring self-reported fatigue among older people. *Qual. Life. Res.* **24**, 2239–2255 (2015). 25778536 10.1007/s11136-015-0963-1

[22] Kratz, A. L. et al. Development of a person-centered conceptual model of perceived fatigability. *Qual. Life. Res.* **28**, 1337–1347 (2019). 30604341 10.1007/s11136-018-2093-z PMC7395299

[23] Page, M. J. et al. The prisma 2020 statement: an updated guideline for reporting systematic reviews. *Bmj.* **372**, n71 (2021). 33782057 10.1136/bmj.n71 PMC8005924

[24] Stang, A. Critical evaluation of the newcastle-ottawa scale for the assessment of the quality of nonrandomized studies in meta-analyses. *Eur. J. Epidemiol.* **25**, 603–605 (2010). 20652370 10.1007/s10654-010-9491-z

[25] Borenstein, M., Hedges, L. V., Higgins, J. P. & Rothstein, H. R. A basic introduction to fixed-effect and random-effects models for meta-analysis. *Res. Synth. Methods.* **1**, 97–111 (2010). 26061376 10.1002/jrsm.12

[26] Higgins, J. P., Thompson, S. G., Deeks, J. J. & Altman, D. G. Measuring inconsistency in meta-analyses. *Bmj.* **327**, 557–560 (2003). 12958120 10.1136/bmj.327.7414.557 PMC192859

[27] Higgins, J. P. & Thompson, S. G. Quantifying heterogeneity in a meta-analysis. *Stat. Med.* **21**, 1539–1558 (2002). 12111919 10.1002/sim.1186

[28] Sterne, J. A. et al. Statistical methods for assessing the influence of study characteristics on treatment effects in “meta-epidemiological” research. *Stat. Med.* **21**, 1513–1524 (2002). 12111917 10.1002/sim.1184

[29] Glynn, N. W. et al. Perceived physical fatigability predicts all-cause mortality in older adults. *J. Gerontol. Series A.* **77**, 837–841 (2022). 10.1093/gerona/glab374 PMC8974332 34908118

[30] Alfini, A. J. et al. Associations of actigraphic sleep parameters with fatigability in older adults. *J. Gerontol.: Series A.* **75**, e95–e102 (2020). 10.1093/gerona/glaa137 PMC7494020 32502253

[31] Cooper, R. et al. Are bmi and inflammatory markers independently associated with physical fatigability in old age?. *Int. J. Obes.* **43**, 832–841 (2019). 10.1038/s41366-018-0087-0 PMC6477893 29795469

[32] Pérez, L. M. et al. Validation of the Spanish version of the pittsburgh fatigability scale for older adults. *Aging Clin. Exp. Res.* **31**, 209–214 (2019). 29736892 10.1007/s40520-018-0959-0 PMC6222011

[33] Egerton, T., Chastin, S. F., Stensvold, D. & Helbostad, J. L. Fatigue may contribute to reduced physical activity among older people: an observational study. *J. Gerontol. Ser. A-Biol. Sci. Med. Sci.* **71**, 670–676 (2016). 26347508 10.1093/gerona/glv150

[34] Tennant, K. F., Takacs, S. E., Gau, J. T., Clark, B. C. & Russ, D. W. A preliminary study of symptomatic fatigue in rural older adults. *Aging Clin. Exp. Res.* **24**, 324–330 (2012). 22027409 10.3275/8054

[35] Hu, Y. et al. Validation of perceived physical fatigability using the simplified-chinese version of the pittsburgh fatigability scale. *Bmc Geriatr.*10.1186/s12877-021-02275-x (2021). 34039260 10.1186/s12877-021-02275-x PMC8157666

[36] Banerjee, N. et al. Structural basal ganglia correlates of subjective fatigue in middle-aged and older adults. *J. Geriatr. Psychiatry. Neurol.*10.1177/08919887211070264 (2022). 35202547 10.1177/08919887211070264

[37] Malak, M. Z., Abu, A. M., Al-Amer, R., Yousef, N. N. & Ali, R. M. Evaluation of fatigue among older population in Jordan. *Exp. Aging Res.* **47**, 464–477 (2021). 33792513 10.1080/0361073X.2021.1908764

[38] Blain, H. et al. Self-reported fatigue: a significant risk factor for falling in older women and men. *Exp. Gerontol.* **143**, 111154 (2021). 33189836 10.1016/j.exger.2020.111154

[39] Cho, J. H. et al. Associations of objective versus subjective social isolation with sleep disturbance, depression, and fatigue in community-dwelling older adults. *Aging Ment. Health.* **23**, 1130–1138 (2019). 30284454 10.1080/13607863.2018.1481928 PMC6447478

[40] LaSorda, K. R. et al. Epidemiology of perceived physical fatigability in older adults: the long life family study. *J. Gerontol. Series A.* **75**, e81–e88 (2020). 10.1093/gerona/glz288 PMC7494027 31828303

[41] Başkurt, Z., Başkurt, F. & Demir, C. The effects of self-perceived fatigue on functional mobility and balance in the community-dwelling elderly. *Healthmed.* **6**, 3366–3371 (2012).

[42] Cohen, R. W. et al. Prevalence and severity of perceived mental fatigability in older adults: the long life family study. *J. Am. Geriatr. Soc.* **69**, 1401–1403 (2021). 33675035 10.1111/jgs.17075 PMC8142668

[43] Qiao, Y. S. et al. Changes in objectively measured physical activity are associated with perceived physical and mental fatigability in older men. *J. Gerontol.: Series A.* **12**, 2507–2516 (2022). 10.1093/gerona/glac082 PMC9799193 35385877

[44] Schnelle, J. F. et al. Evaluation of Two Fatigability Severity Measures in Elderly Adults. *J. Am. Geriatr. Soc.* **60**, 1527–1533 (2012). 22860899 10.1111/j.1532-5415.2012.04062.x PMC3419324

[45] Moored, K. D. et al. Life-space mobility in older men: the role of perceived physical and mental fatigability. *J. Gerontol: Series A.* **60**, 1527–1533 (2021). 10.1093/gerona/glab286 PMC9678195 34718553

[46] Graves, J. L. et al. Profiles of accelerometry-derived physical activity are related to perceived physical fatigability in older adults. *Sensors.* **21**, 1718 (2021). 33801352 10.3390/s21051718 PMC7958607

[47] Wasson, E. et al. Neural correlates of perceived physical and mental fatigability in older adults: a pilot study. *Exp. Gerontol.* **115**, 139–147 (2019). 30528639 10.1016/j.exger.2018.12.003 PMC6331252

[48] Davis, B. et al. The association between poor diet quality, physical fatigability and physical function in the oldest-old from the geisinger rural aging study. *Geriatrics.* **6**, 41 (2021). 33920900 10.3390/geriatrics6020041 PMC8167721

[49] Renner, S. W. et al. Validation of perceived mental fatigability using the pittsburgh fatigability scale. *J. Am. Geriatr. Soc.* **69**, 1343–1348 (2021). 33469914 10.1111/jgs.17017 PMC8127403

[50] Palmberg, L. et al. The associations of activity fragmentation with physical and mental fatigability among community-dwelling 75-, 80-, and 85-year-old people. *The Journals of Gerontology: Series A.* **75**, e103–e110 (2020). 10.1093/gerona/glaa166 32614396

[51] Flensner, G., Ek, A. C., Landtblom, A. M. & Söderhamn, O. Fatigue in relation to perceived health: people with multiple sclerosis compared with people in the general population. *Scand. J. Caring Sci.* **22**, 391–400 (2008). 18840223 10.1111/j.1471-6712.2007.00542.x

[52] Jamal, M. H., Abdul, A. A., Aizuddin, A. N. & Aljunid, S. M. Successes and obstacles in implementing social health insurance in developing and middle-income countries: a scoping review of 5-year recent literatures. *Front. Public Health.* **10**, 918188 (2022). 36388320 10.3389/fpubh.2022.918188 PMC9648174

[53] Docrat, S., Besada, D., Cleary, S. & Lund, C. The impact of social, national and community-based health insurance on health care utilization for mental, neurological and substance-use disorders in low- and middle-income countries: a systematic review. *Health Econ. Rev.* **10**, 11 (2020). 32333114 10.1186/s13561-020-00268-x PMC7181535

[54] Araja, D., Berkis, U., Lunga, A. & Murovska, M. Shadow burden of undiagnosed myalgic encephalomyelitis/chronic fatigue syndrome (Me/Cfs) on society: retrospective and prospective—in light of covid-19. *J. Clin. Med.* **10**, 3017 (2021). 34300183 10.3390/jcm10143017 PMC8303374

[55] Baetz, M. & Bowen, R. Chronic pain and fatigue: associations with religion and spirituality. *Pain Res. Manag.* **13**, 383–388 (2008). 18958309 10.1155/2008/263751 PMC2799261

[56] Keller-Ross, M. L., Larson, M. & Johnson, B. D. Skeletal muscle fatigability in heart failure. *Front. Physiol.* **10**, 129 (2019). 30846944 10.3389/fphys.2019.00129 PMC6393404

[57] Zhang, S. et al. Related risk factors associated with post-stroke fatigue: a systematic review and meta-analysis. *Neurol. Sci.* **42**, 1463–1471 (2021). 32813167 10.1007/s10072-020-04633-w

[58] Association, A. H. *F.a.S.T.: Arm Weakness*. (2024) Available at: https://www.stroke.org/en/fast-experience#.

[59] Whitehead, L. C., Unahi, K., Burrell, B. & Crowe, M. T. The experience of fatigue across long-term conditions: a qualitative meta-synthesis. *J. Pain. Symptom. Manage.* **52**, 131–143 (2016). 27233142 10.1016/j.jpainsymman.2016.02.013

[60] Torossian, M. & Jacelon, C. S. Chronic illness and fatigue in older individuals: a systematic review. *Rehabil. Nurs.* **46**, 125–136 (2021). 32657851 10.1097/RNJ.0000000000000278 PMC7935454

[61] Ho, L. & Ng, S. Non-pharmacological interventions for fatigue in older adults: a systematic review and meta-analysis. *Age. Ageing.* **49**, 341–351 (2020). 32101281 10.1093/ageing/afaa019

[62] Lin, F., Chen, D., Vance, D. E., Ball, K. K. & Mapstone, M. Longitudinal Relationships Between Subjective Fatigue, Cognitive Function, and Everyday Functioning in Old Age. *Int. Psychogeriatr.* **25**, 275–285 (2013). 23083533 10.1017/S1041610212001718 PMC3552486

