# Supplementary material for: Prevalence of fatigue and perceived fatigability in older adults: a systematic review and meta-analysis
Source: Sci Rep. 2025 Feb 9;15:4818. doi: 10.1038/s41598-025-88961-x (PMC11808098; doi:10.1038/s41598-025-88961-x)

Supplementary Material

eMethods 1.Search strategy used in the current systematic review and meta-analysis.

Pubmed

#1 older[Mesh]
#2 Elderly[Title/Abstract] OR old age[Title/Abstract] OR older[Title/Abstract] OR geriatrics[Title/Abstract] OR aged[Title/Abstract] OR ageing[Title/Abstract]
#3 (#1 OR #2)
#4 "fatigue"[Mesh]
#5 fatigue[Title/Abstract] OR tired*[Title/Abstract] OR wear*[Title/Abstract] OR exhaust*[Title/Abstract] OR lackluster[Title/Abstract] OR ((astenia[Title/Abstract] OR asthenic[Title/Abstract]) AND syndrome[Title/Abstract]) OR ((lack[Title/Abstract] OR loss[Title/Abstract] OR lost[Title/Abstract]) near/3 (energy[Title/Abstract] OR vigour[Title/Abstract]))
#6 (#4 OR #5)
#7 Prevalence[Mesh] OR Epidemiology[Mesh] OR Cross-Sectional Studies[Mesh]
#8 prevalence*[Title/Abstract] OR epidemiology[Title/Abstract] OR incidence*[Title/Abstract] OR morbidity[Title/Abstract] OR cross sectional stud*[Title/Abstract] OR cross sectional analys*[Title/Abstract] OR cross sectional survey[Title/Abstract]
#9 (#7 OR #8)
#10 (#3 AND #6 AND #9)

Web of Science

#1 TS=(elderly OR elder OR old age OR older OR geriatrics OR aged OR ageing OR age*)
#2 TS=(fatigue* OR asthenia OR asthenic OR astheni* OR exhaustion OR exhausted OR loss of energy OR loss of vitality OR weary OR weariness OR weakness OR apathy OR apathetic OR lassitude OR lethargic OR lethargy OR sleepy OR sleepiness OR drowsy OR drowsiness OR tired OR tiredness OR energy loss OR vitality loss)
#3 TS=(prevalence* OR epidemiology OR incidence* OR morbidity OR cross sectional stud* OR cross sectional analys* OR cross sectional survey)
#4 (#1 AND #2 AND #3)

Embase

S1 (MH "elderly")
S2 (elderly OR elder OR old age OR older OR geriatrics OR aged OR ageing OR age*)
S3 (S1 OR S2)
S4 (MH "fatigue")
S5 (fatigue OR tired* OR wear* OR exhaust* OR lackluster)
S6 (S4 OR S5)

S7 (MH "Prevalence" OR MH "Epidemiology+" OR MH "Cross Sectional Studies")
S8 (prevalence* OR epidemiology OR incidence* OR morbidity OR cross sectional stud* OR cross sectional analys* OR cross sectional survey)
S9 (S7 OR S8)
S10 (S3 AND S6 AND S9)

Cochrane
#1 MeSH descriptor: [elderly] explode all trees
#2 Elderly OR Elder OR old age OR older OR geriatrics OR aged OR ageing OR age*
#3 (#1 OR #2)
#4 MeSH descriptor: [fatigue] explode all trees
#5 fatigue OR tired* OR wear* OR exhaust* OR lackluster
#6 (#4 OR #5)
#7 MeSH descriptor: [Prevalence] explode all trees OR MeSH descriptor: [Epidemiology] explode all trees OR MeSH descriptor: [Cross-Sectional Studies] explode all trees
#8 prevalence* OR epidemiology OR incidence* OR morbidity OR cross sectional stud* OR cross sectional analys* OR cross sectional survey
#9 (#7 OR #8)
#10 (#3 AND #6 AND #9)

eMethods 2. Modified Newcastle-Ottawa risk of bias scoring guide.

(1) Representativeness of the sample:
1 point: Population contained both urban and rural populations in several regions.
0 points: Population contained the urban or rural population of an area.

(2) Sample size:
1 point: Sample size was greater than 200 participants.
0 points: Sample size was less than 200 participants or a convenience sample.

(3) Non-respondents:
1 point: Comparability between respondent and non-respondent characteristics was established, and the response rate was satisfactory.
0 points: The response rate was unsatisfactory, the comparability between respondents and non-respondents was unsatisfactory, or there was no description of the response rate or the characteristics of the responders and the non-responders.

(4) Ascertainment of fatigue:
1 point: Validated measurement tool using a validated cutoff score.
0 points: Non-validated measurement tool, or validated measurement tool with non-valid cutoff score.

(5) Quality of descriptive statistics reporting:
1 point: Reported descriptive statistics to describe the population (e.g., age, sex) with proper measures of dispersion (e.g., standard deviation, standard error, range).
0 points: Descriptive statistics were not reported, were incomplete, or did not include proper measures of dispersion.

Legend: This scale, the scoring of which ranges from 0 to 5, assesses quality in several domains: sample representativeness and size, comparability between respondents and non-respondents, ascertainment of fatigue, and statistical quality. Studies were judged to be of low risk of bias (≥3 points) or high risk of bias (<3 points).

eTable 1. Newcastle-Ottawa risk of bias assessment

Source	Representativeness	Size	Comparability	Outcome	Statistics	Total
Glynn et al. 2022	1	1	1	1	1	5
Alfini et al. 2020	1	1	0	1	1	4
Simonsick et al. 2018	1	1	0	1	0	3
Cooper et al. 2019	1	1	1	1	1	5
Pérez et al. 2019	0	0	1	1	1	3
Egerton et al. 2016	0	1	1	1	1	4
Tennant et al. 2012	1	0	0	1	0	2
Hu et al. 2021	0	1	1	1	1	4
Banerjee et al. 2022	1	1	1	1	1	5
Malak et al. 2021	1	1	1	1	1	5
Blain et al. 2021	0	1	1	1	1	4
Cho et al. 2019	1	1	1	1	1	5
LaSorda et al. 2020	1	1	1	1	1	5
Başkurt et al. 2012	0	0	0	1	1	2
Cohen et al. 2021	1	1	1	1	1	5
Qiao et al. 2022	1	1	1	1	1	5
Schnelle et al. 2012	0	0	1	1	1	3
Moored et al. 2021	1	1	1	1	1	5
Graves et al. 2021	0	0	1	1	1	3
Wasson et al. 2019	0	0	1	1	1	3
Davis et al. 2021	0	0	1	1	1	3

eFig. 1. Funnel plot of studies assessing fatigue

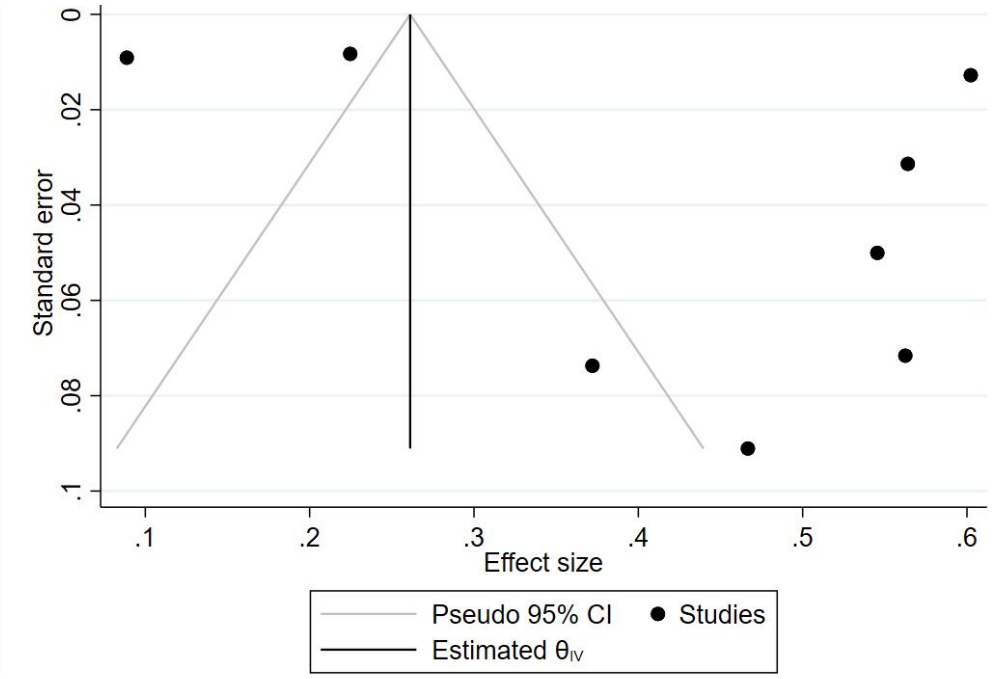

eFig. 2. Funnel plot of studies assessing perceived physical fatigability

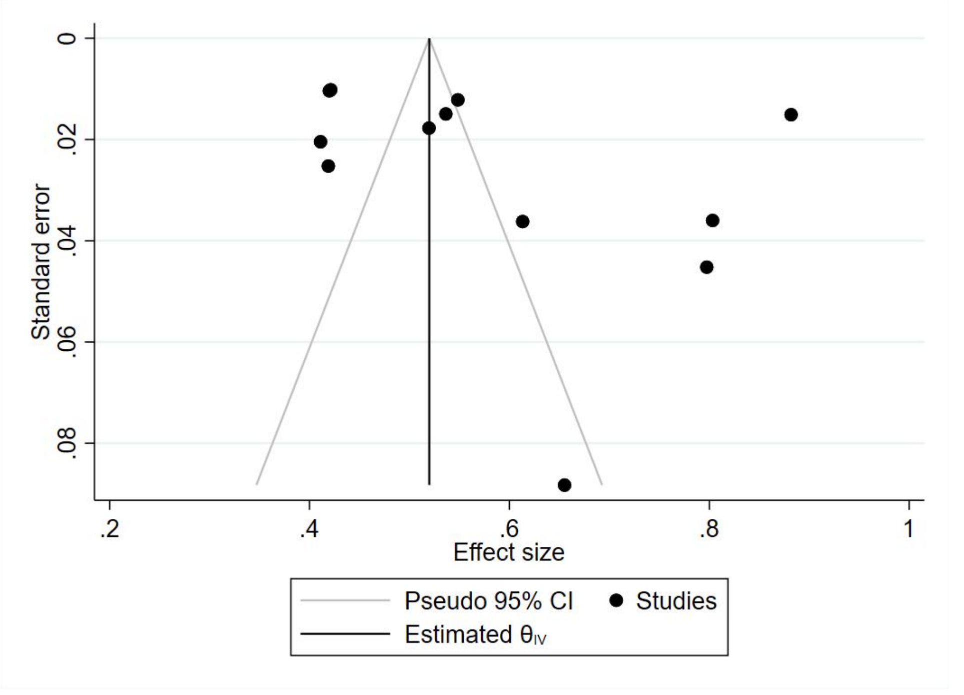

eFig. 3. Funnel plot of studies assessing perceived mental fatigability

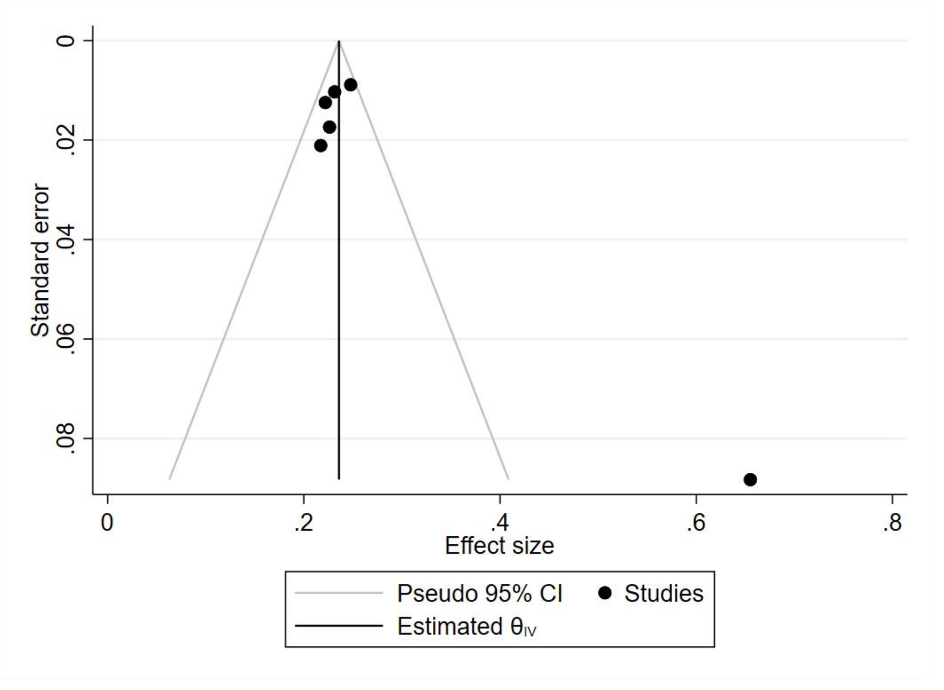

Supplement: Supplementary file 1 — Supplementary Information. [file 41598_2025_88961_MOESM1_ESM.pdf]
